# Diagnosis and clinical outcomes of extrapulmonary tuberculosis in antiretroviral therapy programmes in low‐ and middle‐income countries: a multicohort study

**DOI:** 10.1002/jia2.25392

**Published:** 2019-09-11

**Authors:** Kathrin Zürcher, Marie Ballif, Sasisopin Kiertiburanakul, Henri Chenal, Marcel Yotebieng, Beatriz Grinsztejn, Denna Michael, Timothy R Sterling, Kapella M Ngonyani, Anna M Mandalakas, Matthias Egger, April C Pettit, Lukas Fenner, the International Epidemiology Databases to Evaluate AIDS (IeDEA) consortium, Valdilea Veloso, Paula Luz, Raquel de Boni, Sandra Cardoso Wagner, Ruth Friedman, Ronaldo Moreira, Juan Sierra Madero, Brenda Crabtree Ramirez, Paco Belaunzaran, Yanink Caro Vega, Eduardo Gotuzzo, Fernando Mejia, Gabriela Carriquiry, Catherine C McGowan, Bryan E Shepherd, Timothy Sterling, Karu Jayathilake, Anna K Person, Peter F Rebeiro, Mark Giganti, Jessica Castilho, Stephany N Duda, Fernanda Maruri, Hilary Vansell, E Uy, R Bantique, A vihingsanon, S Gatechompol, P Phanuphak, C Phadungphon, S Kiertiburanakul, A Phuphuakrat, L Chumla, N Sanmeema, KV Nguyen, HV Bui, DTH Nguyen, DT Nguyen, DD Cuong, NV An, NT Luan, AH Sohn, JL Ross, B Petersen, DA Cooper, MG Law, A Jiamsakul, DC Boettiger, John Ssali, Mathew Ssemakadde, Kapella Ngonyani, Jerome Lwali, Mark Urassa, Richard Machemba, Kara Wools‐Kaloustian, Constantin Yiannoutsos, Rachel Vreeman, Beverly Musick, Batya Elul, Rami Kantor, Jeffrey Martin, Megan Wenger, Craig Cohen, Jayne Kulzer, Djimon Marcel Zannou, Angèle Azon‐Kouanou, Hamar Alassane Traore, Daouda Minta, Amadou Abathina Toure, Moussa Seydi, Coumba Cissé Bassabi, François Dabis, Emmanuel Bissagnene, Elise Arrivé, Patrick Coffie, Didier Ekouevi, Antoine Jaquet, Valériane Leroy, Charlotte Lewden, Annie J Sasco, Dieudonné Amani, Jean‐Claude Azani, Eric Balestre, Serge Bessekon, Franck Bohossou, Camille Gilbert, Sophie Karcher, Jules Mahan Gonsan, Jérôme Le Carrou, Séverin Lenaud, Célestin Nchot, Karen Malateste, Amon Roseamonde Yao, Bertine Siloué, Gwenaelle Clouet, Madikona Dosso, Alexandra Doring, Adrienne Kouakou, Elodie Rabourdin, Jean Rivenc, Xavier Anglaret, Boubacar Ba, Andrea Ciaranello, Sébastien Datté, Sophie Desmonde, Jean‐Serge Elvis Diby, Geoffrey S Gottlieb, Serge N'zoré Kangah, Denis Malvy, David Meless, Aida Mounkaila‐Harouna, Camille Ndondoki, Boris Tchounga, Rodolphe Thiébaut, Gilles Wandeler, Jean Claude Dusingize, Eugene Mutimura, Eugene Mutimura, Judy Tatwangire, Izimukwiye Izabelle, Evelyne Baramperanye, Andrew Edmonds, Innocent Azinyue, Liliane Ayangma

**Affiliations:** ^1^ Institute of Social and Preventive Medicine (ISPM) University of Bern Bern Switzerland; ^2^ Faculty of Medicine Ramathibodi Hospital Mahidol University Bangkok Thailand; ^3^ Centre Intégré de Recherches Biocliniques d'Abidjan (CIRBA) Abidjan Côte d'Ivoire; ^4^ College of Public Health The Ohio State University Columbus OH USA; ^5^ Instituto Nacional de Infectologia Evandro Chagas Fundação Oswaldo Cruz Rio de Janeiro Brazil; ^6^ National Institute for Medical Research Kisesa HDSS Mwanza Tanzania; ^7^ Vanderbilt Tuberculosis Center Nashville TN USA; ^8^ Division of Infectious Diseases Vanderbilt University Medical Center Nashville TN USA; ^9^ Tumbi Special Hospital CTC Kibaha Town Tanzania; ^10^ The Global Tuberculosis Program Texas Children’s Hospital and Baylor College of Medicine Houston TX USA; ^11^ Centre for Infectious Disease Epidemiology & Research School of Public Health & Family Medicine University of Cape Town South Africa

**Keywords:** extrapulmonary tuberculosis, pulmonary tuberculosis, tuberculosis, HIV‐positive patients, low‐ and middle‐income countries, diagnostics, mortality, lost to follow‐up

## Abstract

**Introduction:**

Extrapulmonary tuberculosis (EPTB) is difficult to confirm bacteriologically and requires specific diagnostic capacities. Diagnosis can be especially challenging in under‐resourced settings. We studied diagnostic modalities and clinical outcomes of EPTB compared to pulmonary tuberculosis (PTB) among HIV‐positive adults in antiretroviral therapy (ART) programmes in low‐ and middle‐income countries (LMIC).

**Methods:**

We collected data from HIV‐positive TB patients (≥16 years) in 22 ART programmes participating in the International Epidemiology Databases to Evaluate AIDS (IeDEA) consortium in sub‐Saharan Africa, Asia‐Pacific, and Caribbean, Central and South America regions between 2012 and 2014. We categorized TB as PTB or EPTB (EPTB included mixed PTB/EPTB). We used multivariable logistic regression to assess associations with clinical outcomes.

**Results and Discussion:**

We analysed 2695 HIV‐positive TB patients. Median age was 36 years (interquartile range (IQR) 30 to 43), 1102 were female (41%), and the median CD4 count at TB treatment start was 114 cells/μL (IQR 40 to 248). Overall, 1930 had PTB (72%), and 765 EPTB (28%). Among EPTB patients, the most frequently involved sites were the lymph nodes (24%), pleura (15%), abdomen (11%) and meninges (6%). The majority of PTB (1123 of 1930, 58%) and EPTB (582 of 765, 76%) patients were diagnosed based on clinical criteria. Bacteriological confirmation (using positive smear microscopy, culture, Xpert MTB/RIF, or other nucleic acid amplification tests result) was obtained in 897 of 1557 PTB (52%) and 183 of 438 EPTB (42%) patients. EPTB was not associated with higher mortality compared to PTB (adjusted odd ratio (aOR) 1.0, 95% CI 0.8 to 1.3), but TB meningitis was (aOR 1.9, 95% CI 1.0 to 3.1). Bacteriological confirmation was associated with reduced mortality among PTB patients (aOR 0.7, 95% CI 0.6 to 0.8) and EPTB patients (aOR 0.3 95% CI 0.1 to 0.8) compared to TB patients with a negative test result.

**Conclusions:**

Diagnosis of EPTB and PTB at ART programmes in LMIC was mainly based on clinical criteria. Greater availability and usage of TB diagnostic tests would improve the diagnosis and clinical outcomes of both EPTB and PTB.

## 1 Introduction

In low‐ and middle‐income countries (LMIC), tuberculosis (TB) accounts for approximately 40% of HIV/AIDS‐related deaths among adults, and half of those TB cases are undiagnosed at the time of death 1. TB predominantly affects the lungs (pulmonary tuberculosis [PTB]), but can affect extrapulmonary sites as well (EPTB). Globally, about 25% of all TB cases are estimated to be EPTB 2. EPTB is a common presentation in HIV‐positive individuals, particularly in those with low CD4 cell counts 3, 4. EPTB is most frequently identified in the lymph nodes, pleura, bones and joints, abdomen, meninges and genitourinary tract 5. TB meningitis is considered the most severe form of EPTB with mortality as high as 70% in low‐income countries 6.

The diagnosis of EPTB is particularly difficult and is often solely based on clinical signs and symptoms. A bacteriological confirmation of EPTB often requires invasive specimen collection by biopsy or fine needle aspiration 7, 8, followed by use of adequate diagnostic tests 9, 10. Much progress has been made in developing new diagnostic tests for TB, including the Xpert MTB/RIF and other nucleic acid amplification tests (NAAT), which have higher sensitivity than smear microscopy and can also be used to diagnose both PTB and EPTB 11. This study assessed diagnostic modalities and TB treatment outcomes of EPTB compared to PTB in HIV‐positive adults in clinical care in antiretroviral therapy (ART) programmes in six International epidemiology Databases to Evaluate AIDS (IeDEA) regions in sub‐Saharan Africa, Asia‐Pacific, Caribbean, Central and South America Central and South America.

## 2 Methods

### 2.1 Study setting and study population

IeDEA ( www.iedea.org) is a large consortium of ART programmes predominantly located in LMIC 12. ART programmes in six IeDEA regions participated in this study are mostly public but often supported by NGOs or academia: East Africa; Central Africa; West Africa; Southern Africa; Asia‐Pacific; Caribbean, Central and South America.

**Figure 1:**
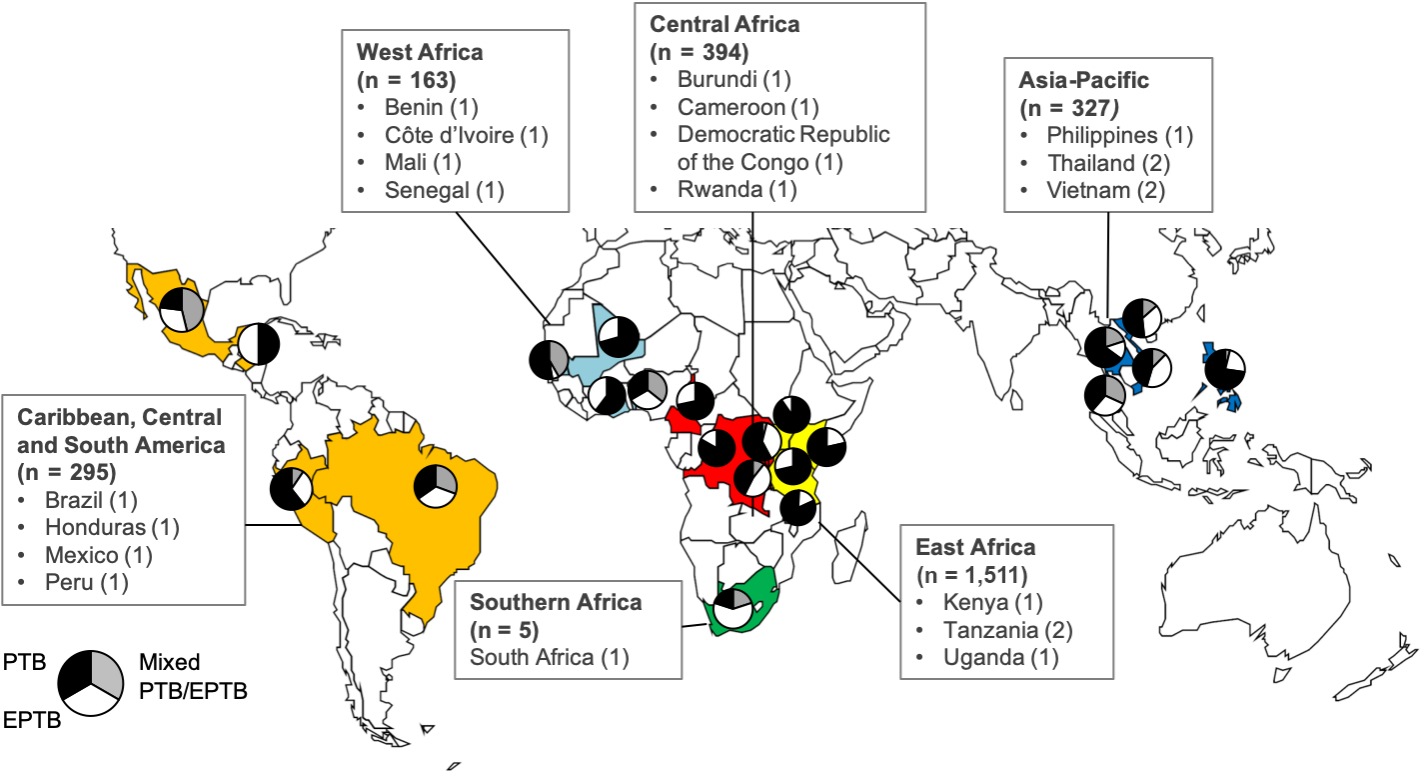
Geographical distribution of 22 antiretroviral treatment (ART) programmes treating HIV‐positive patients (≥16 years) in low‐ and middle‐income countries. The proportions of pulmonary (black), mixed pulmonary/extrapulmonary (grey), and extrapulmonary tuberculosis cases (white) diagnosed at each site are indicated in the pie charts; n indicates the number of patients included in the study by IeDEA region; the numbers in parentheses following the country names indicate the number of ART programmes participating in the study by country. EPTB, extrapulmonary tuberculosis; IeDEA, International Epidemiology Databases to Evaluate AIDS; PTB, pulmonary tuberculosis.

We reviewed records of consecutive 3165 HIV‐positive patients diagnosed with any form of TB between January 1, 2012 and December 31, 2014 in participating ART programmes. Patient records missing data on sex, date of birth or site of disease were excluded from the analysis (35 records). We studied only adults and excluded paediatric cases (age <16 years, 396 records). In case of multiple TB episodes, only the patient's first episode was included in the study (39 duplicate records deleted). This resulted in the inclusion of 2695 adult HIV‐positive TB patients from 22 ART programmes (Figure 1).

### 2.2 Data collection

Standardized electronic case report forms (CRFs), available in English or French, were used to record age, sex, date of TB diagnosis, site of TB disease, site of EPTB manifestation (predominant organ), start date of TB treatment, body mass index (BMI) at start of TB treatment, ART status at TB diagnosis, CD4 cell count at start of TB treatment, previous history of TB, TB drug resistance, results from TB diagnostic tests (smear microscopy, culture, Xpert MTB/RIF and other NAAT) and TB treatment outcomes 13. All data were collected using REDCap ( www.project-redcap.org) 14. Local IeDEA site investigators completed CRFs for TB patients. Data were entered between January 2012 and January 2016. During data collection, routine audits were made to ensure data quality 15. Furthermore, we used programme‐level data previously collected in the same ART programmes in 2012 16.

### 2.3 Definitions

We categorized TB as PTB (involving the lungs only), mixed PTB/EPTB and EPTB only 5. For binary outcome analyses, we used the categories PTB and EPTB (includes mixed PTB/EPTB cases) as previously defined 17. Miliary TB was categorized as EPTB. Furthermore, we categorized bacteriological confirmation as “test performed” if at least one of the tests (smear microscopy, culture, Xpert MTB/RIF and/or other NAATs) was performed regardless of whether the result was positive or negative; “positive” bacteriological confirmation (any positive test result); “negative” (all performed tests were negative); and “no test performed.” TB treatment outcomes were categorized as cured, treatment completed, treatment failed, died, lost to follow‐up (LTFU) and not evaluated 5. The category “not evaluated” included patients who were still on treatment, transferred out and those whose treatment outcome was unknown. The category “treatment success” included cured patients and patients who completed TB treatment.

### 2.4 Statistical analyses

We used descriptive statistics to characterize both programme‐level and patient‐level data, as well as diagnostic modalities. Differences between groups were assessed using chi‐square, Fisher's exact or Wilcoxon rank‐sum tests as appropriate. We used univariate and multivariate logistic regressions to assess risk factors for mortality and LTFU during TB treatment. These associations were presented as unadjusted odds ratios (ORs) and ORs adjusted (aORs) for age, sex, BMI at start of TB treatment, previous history of TB, ART status at TB diagnosis and CD4 cell counts at start of TB treatment, taking into account heterogeneity across regions (clustering by treatment programmes). In separate models, we obtained the estimates for EPTB sites, (adjusted for the same co‐variates). Patients without documented treatment outcomes were excluded from the primary analysis. However, we performed a sensitivity analysis considering patients LTFU as having died. To account for missing data we used multiple imputations by chained equations to impute missing BMI, ART status at TB diagnosis, CD4 cell counts at start of TB treatment and previous history of TB. The quality of the imputation can be improved by adding variables outside the analysis 18, therefore in addition to the outcome and the covariates used in the analysis, we also considered the date of TB treatment start, IeDEA region, setting, level of care for imputation. We ran the model on 20 imputed datasets for each analysis and used the Rubin rule to pool the estimates. All analyses were performed in STATA (version 14.1, Stata Corporation, Texas, USA).

### 2.5 Ethics statement

Local institutional review board or ethics committee approval was obtained at all local study sites. Informed consent was obtained where requested per local regulations. The Vanderbilt University Medical Center Institutional Review Board, Nashville, Tennessee (USA), and the Cantonal Ethics Committee Bern (Switzerland) approved the analyses for this specific project.

## 3 Results and discussion

### 3.1 Study sites and patient characteristics

The 2695 HIV‐positive TB patients participating in this study were treated at 22 ART programmes in 19 countries (Figure 1). Eighteen sites were urban, three were peri‐urban and one site was rural. The level of care was mostly tertiary, at 17 sites, followed by secondary at four sites and primary at one site. For TB diagnostics a free‐of‐charge cost model was available at 11 of the 22 sites, a cost sharing model was available at 10 of the 22 sites and one site had a mixed cost model.

The median patient age was 35.5 years (interquartile range (IQR) 29.9 to 42.8), and 1102 patients (40.9%) were female. Among the 2695 patients, 1930 had PTB (71.6%) and 765 had EPTB (28.4%); 131 patients (4.9% overall) had both. At the time of TB diagnosis, 1270/2965 (47.1%) TB patients had not started ART and 763/2695 (28.3%) TB patients were on ART; the ART status of the remaining patients was unknown. Of the TB patients on ART, 342/763 (44.8%) were more than six months on ART before TB diagnosis and 421/763 (55.2%) were six or less months on ART. Among the 765 EPTB patients, the most frequent sites of disease were the lymph nodes (24.4%), pleura (14.2%), abdomen (11.1%), and meninges (6.3%). Complete patient characteristics are given in Table 1. When stratifying CD4 cell counts at the time of TB treatment (0 to 49 cells/μL; 50 to 199 cells/μL; ≥200 cells/μL; missing CD4 values), the frequencies of sites of EPTB manifestations remained similar over all groups.

**Table 1:** Characteristics of HIV‐positive patients diagnosed with pulmonary tuberculosis and extrapulmonary tuberculosis at the start of TB treatment in 22 antiretroviral treatment programmes from lower income countries

	All	PTB	EPTB		
			EPTB	Mixed PTB/EPTB	EPTB only
Total, n (%)	2695 (100)	1930 (71.6)	765 (28.4)	131 (4.9)	634 (23.5)
Age, year					
16 to 29	588 (21.8)	414 (21.4)	174 (22.8)	28 (21.4)	146 (23.0)
30 to 39	1105 (41.0)	793 (41.1)	312 (40.8)	59 (45.0)	253 (39.9)
40 to 49	696 (25.8)	503 (26.1)	193 (25.2)	35 (26.7)	158 (24.9)
50	306 (11.4)	220 (11.4)	86 (11.2)	9 (6.9)	77 (12.2)
Sex, n (%)					
Male	1593 (59.1)	1134 (58.8)	459 (60.0)	90 (68.7)	369 (58.2)
Female	1102 (40.9)	796 (41.2)	306 (40.0)	41 (31.3)	265 (41.8)
BMI at start of TB treatment kg/m^2^, median (IQR)	18.7 (16.8 to 20.9)	18.6 (16.7 to 20.8)	18.8 (16.9 to 21.1)	18.1 (16.4 to 19.8)	18.9 (17.0 to 21.4)
*No. of observations (%)*	2115 (78.5)	1553 (80.5)	562 (73.5)	82 (62,6)	480 (75.7)
CD4 count at TB treatment start, median (IQR), cells/μL	114 (40 to 248)	124 (45 to 263)	92 (32 to 212)	55 (19 to 129)	105 (34 to 228)
*No. of observations (%)*	2196 (81.5)	1575 (81.6)	621 (81.2)	108 (82.4)	513 (80.9)
ART status at TB diagnosis					
Not on ART	1270 (47.1)	936 (48.5)	334 (43.7)	51 (38.9)	283 (44.6)
On ART	763 (28.3)	599 (31.0)	164 (21.4)	12 (9.2)	152 (24.0)
6 + months at TB diagnosis	342 (12.7)	268 (13.9)	74 (9.7)	4 (3.1)	70 (11.0)
<6 months at TB diagnosis	421 (15.6)	331 (17.2)	90 (11.8)	8 (6.1)	82 (12.9)
Missing	662 (24.7)	395 (20.5)	267 (34.9)	68 (51.9)	199 (31.4)
Previous history of TB, n (%)					
Yes	55 (2.0)	37 (1.9)	18 (2.4)	3 (2.3)	15 (2.4)
No	2304 (85.5)	1705 (88.3)	599 (78.3)	82 (62.6)	517 (81.5)
Unknown	336 (12.5)	188 (9.7)	148 (19.3)	46 (35.1)	102 (16.1)
TB treatment outcomes, n (%)					
Treatment success^a^	1908 (70.8)	1383 (71.7)	525 (68.6.9)	75 (57.3)	450 (71.0)
Treatment failed	15 (0.6)	13 (0.7)	2 (0.3)	1 (0.8)	1 (0.2)
Died	281 (10.4)	194 (10.1)	87 (11.4)	13 (9.9)	74 (11.7)
Lost to follow‐up (default)	136 (5.0)	100 (5.2)	36 (3.2)	16 (12.2)	20 (3.2)
Not evaluated^b^	355 (13.2)	240 (12.4)	113 (14.0)	24 (18.3)	89 (14.0)
Organs involved in EPTB, n (%)					
Lymph nodes^c^	187 (6.9)	‐	187 (24.4)	31 (23.7)	156 (24.6)
Pleura	109 (4.0)	‐	109 (14.2)	15 (11.5)	94 (14.8)
Abdomen	85 (3.2)	‐	85 (11.1)	13 (9.9)	72 (11.4)
Meninges	48 (1.8)	‐	48 (6.3)	10 (7.6)	38 (6.0)
Miliary^d^	32 (1.2)	‐	32 (4.2)	10 (7.6)	22 (3.5)
Joints and/or bones	22 (0.8)	‐	22 (2.9)	4 (3.1)	18 (2.8)
Pericardium	13 (0.5)	‐	13 (1.7)	3 (2.3)	10 (1.6)
Genitourinary tract	3 (0.1)	‐	3 (0.4)	1 (0.8)	2 (0.3)
Larynx	1 (<0.1)	‐	1 (0.1)	‐	1 (0.2)
Unknown	265 (9.8)	‐	265 (34.6)	44 (33.6)	221 (34.9)
IeDEA region, n (%)					
Caribbean/C‐S America	295 (11.0)	160 (8.3)	135 (17.6)	42 (32.1)	93 (14.7)
Asia‐Pacific	327 (12.1)	176 (9.1)	151 (19.7)	45 (34.4)	106 (16.7)
West Africa	163 (6.0)	87 (4.5)	76 (9.9)	30 (22.9)	46 (7.3)
Central Africa	394 (14.6)	262 (13.6)	132 (17.3)	9 (6.9)	123 (19.4)
East Africa	1511 (56.1)	1244 (64.5)	267 (34.9)	4 (3.1)	263 (41.5)
Southern Africa	5 (0.2)	1 (<0.1)	4 (0.5)	1 (0.8)	3 (0.5)

ART, antiretroviral therapy; BMI, body mass index; Caribbean/C‐S America, Caribbean, Central and South America; EPTB, extrapulmonary tuberculosis; IQR, interquartile range; MDR, multidrug‐resistant; n, number; PTB, pulmonary tuberculosis; TB, Tuberculosis.

^a^Treatment success includes cured patients and patients who completed TB treatment; ^b^not evaluated includes on treatment, transfer out, and unknown; ^c^extra‐ and intrathoracic; ^d^miliary TB defined as EPTB.

### 3.2 Diagnostics of EPTB and PTB

Diagnostic capabilities varied according to sites. Sputum smear microscopy was available at all sites. Culture was not available at one site each in East Africa (1/4) and Central Africa (1/4), and Xpert MTB/RIF was not available at half of the sites: two in East Africa (2/4), three in Central Africa (3/4), two in West Africa (2/4), one in Asia‐Pacific (1/5), and three in Caribbean, Central and South America (3/4) and other NAATS were not available in half of the sites: two in East Africa (2/4), two in Central Africa (2/4), two in West Africa (2/4), three in Asia‐Pacific (3/5), and two in Caribbean, Central and South America (2/4). Bacteriological confirmation of PTB, EPTB only, and PTB/EPTB was sought in varying proportions in the three groups, and test results in groups varied as well. A confirmatory bacteriological test was performed in 438 of 765 EPTB (including mixed PTB/EPTB) patients (57.3%) and 1557 of 1930 PTB patients (80.7%). Bacteriological confirmation by any test (positive test result) was obtained in 183 of those 438 EPTB patients (41.8%), 103 of 334 patients with only EPTB (30.8%) and 807 of the 1557 PTB patients (51.8%). The diagnoses of the remaining EPTB and PTB patients who were not tested or whose test results were negative were based on clinical criteria (Table 2).

**Table 2:** Diagnostic testing (smear microscopy, culture, Xpert MTB/RIF and/or nucleic acid amplification tests) of PTB and EPTB in HIV‐positive patients: proportion of bacteriologically confirmed results (any positive result/any confirmatory test performed (positive and negative)), and proportions by specific diagnostic tests (smear microscopy, culture and Xpert MTB/RIF)

	Total	Bacteriological confirmation^a^	Smear microscopy confirmation	Culture confirmation	Xpert MTB/RIF confirmation
	n (%)	Proportion n/n, (%)	Proportion n/n, (%)	Proportion n/n, (%)	Proportion n/n, (%)
Site of disease					
PTB	1930 (100)	807/1557 (51.8)	805/1531 (52.6)	95/191 (49.7)	53/77 (68.8)
EPTB	765 (100)	183/438 (41.8)	118/416 (28.4)	75/133 (56.4)	23/35 (65.7)
EPTB only	634 (100)	103/334 (30.8)	58/317 (18.3)	43/84 (51.2)	12/20 (60.0)
Mixed PTB/EPTB	131 (100)	80/104 (76.9)	60/99 (60.6)	32/49 (65.3)	11/15 (73.3)
Organs involved in EPTB					
Lymph nodes	187 (100)	77/131 (58.8)	46/115 (40.0)	17/32 (53.1)	15/18 (75.0)
Meninges	48 (100)	14/29 (48.3)	5/22 (22.7)	4/7 (57.1)	4/4 (100)
Abdomen	85 (100)	18/58 (31.0)	9/45 (20.0)	3/8 (27.5)	1/1 (100)
Pleura	109 (100)	14/49 (28.6)	7/47 (14.9)	3/6 (50.0)	‐
Joint/bones	22 (100)	6/12 (50.0)	4/11 (36.4)	3/4 (75.0)	0/1 (0)
Miliary	32 (100)	5/20 (25.0)	3/17 (17.6)	5/5 (100)	1/1 (100)
Other	17 (100)	3/10 (30.0	2/9 (22.2)	1/1 (100)	1/1 (100)
Unknown	265 (100)	58/154 (37.7)	42/150 (28.0)	39/70 (55.7)	1/9 (11.1)

EPTB, extrapulmonary tuberculosis; n, numbers; PTB, pulmonary tuberculosis.

a Bacteriological confirmation was defined as confirmed if any diagnostic test result was positive (smear microscopy, culture, Xpert MTB/RIF and/or nucleic acid amplification tests).

Among EPTB patients, smear microscopy was the most frequently performed diagnostic test (in 416 of 765 patients, 54.4%) and Xpert MTB/RIF was the least frequently performed diagnostic test (in 35 of 584 patients, 6.0%), but had the highest proportion of bacteriological confirmation (in 23 of 35 patients, 65.7%). The highest proportion of bacteriological confirmation was found among patients with lymph node TB, (in 77 of 131 patients, 58.8%).

### 3.3 Patient factors associated with LTFU and mortality

In a multivariate model, LTFU during TB treatment was equivalent in EPTB patients’ (including both PTB/EPTB) compared to PTB patients (aOR 0.92, 95% CI 0.36 to 2.32). It was also equivalent in EPTB patients’ only compared to PTB patients (aOR 0.58, 95% CI 0.30 to 1.13). However, patients with both PTB/EPTB had at higher odds for LTFU compared to PTB patients (aOR 2.59, 95% CI 1.06 to 6.32, Table 3). EPTB mortality was similar to that of PTB (aOR 1.03, 95% CI 0.84 to 1.27; Table 3). However, TB meningitis was associated with increased mortality (aOR 1.85, 95% CI 1.00 to 3.10) compared to PTB, and overall mortality was also higher in patients with CD4 cell counts <50 cells/μL compared to those with CD4 cell counts ≥200 cells/μL (aOR 2.60, 95% CI 1.46 to 4.64; Table 3). Sensitivity analyses (Table 3) considering patients LTFU as having died showed similar results. From a separate model, bacteriological confirmation was associated with reduced mortality among PTB patients (aOR 0.68, 95% CI 0.61 to 0.76) and EPTB patients (aOR 0.32 95% CI 0.13 to 0.79) compared to all other TB patients with a negative test result.

**Table 3:** Risk factors for lost to follow‐up and mortality during tuberculosis treatment in HIV‐positive patients diagnosed with extrapulmonary and pulmonary TB

Variable	No. of patients	Lost to follow‐up (LTFU)					Mortality				
	n	No. LTFU (%)	Unadjusted OR (95% CI)	*p*‐value	Adjusted OR (95% CI)	*p*‐value	No. of deaths (%)	Unadjusted OR (95% CI)	*p*‐value	Adjusted OR (95% CI)	*p*‐value
Age, years				0.15		0.007			0.01		<0.001
16 to 29	494	38 (7.7)	1		1		43 (8.7)	1		1	
30 to 39	966	58 (6.0)	0.77 (0.50 to 1.17)		0.79 (0.54 to 1.16)		109 (11.3)	1.33 (0.92 to 1.94)		1.20 (0.85 to 1.71)	
40 to 49	617	29 (4.7)	0.59 (0.36 to 0.97)		0.65 (0.49 to 0.85)		90 (14.6)	1.79. (1.22 to 2.63)		1.80 (0.84 to 3.87)	
≥50	263	11 (4.2)	0.57 (0.29 to 1.12)		0.61 (0.24 to 1.56)		39 (14.8)	1.83 (1.15 to 2.90)		1.95 (1.26 to 3.00)	
Sex				0.39		0.55			0.95		0.68
Female	953	51 (5.4)	1		1		114 (12.0)	1		1	
Male	1387	85 (6.1)	1.17 (0.82 to 1.67)		1.14 (0.74 to 1.75)		167 (12.0)	1.00 (0.78 to 1.29)		0.89 (0.50 to 1.56)	
BMI at start of TB treatment, kg/m^2^	2340	‐	0.91 (0.85 to 0.97)	0.004	0.88 (0.81 to 0.96)	0.003	‐	0.96 (0.93 to 1.01)	0.13	0.98 (0.94 to 1.01)	0.19
History of TB				0.57		0.71			0.47		0.87
No	2293	132 (5.8)	1		1		274 (11.9)	1		1	
Yes	47	4 (8.5)	1.37 (0.47 to 3.94)		1.30 (0.32 to 5.23)		7 (14.9)	1.36 (0.59 to 3.12)		0.91 (0.29 to 2.87)	
ART status at TB diagnosis				0.13		0.20			0.94		0.28
Not on ART	1660	104 (6.3)	1		1		200 (12.0)	1		1	
On ART	680	32 (4.7)	0.73 (0.49 to 1.10)		0.75 (0.48 to 1.17)		81 (11.9)	0.99 (0.75 to 1.30)		1.21 (0.85 to 1.74)	
CD4 count at TB treatment start, cells/μL				0.19		0.024			<0.001		0.008
0 to 49	646	40 (6.2)	1.36 (0.84 to 2.21)		1.26 (0.94 to 1.69)		115 (17.8)	2.27 (1.61 to 3.19)		2.60 (1.46 to 4.64)	
50 to 199	829	59 (7.1)	1.51 (0.97 to 2.34)		1.46 (1.09 to 1.97)		89 (10.7)	1.25 (0.89 to 1.75)		1.38 (0.99 to 1.93)	
≥200	865	39 (4.5)	1		1		77 (8.9)	1		1	
Site of disease				0.82		0.86			0.21		0.75
PTB	1688	100 (5.9)	1		1		194 (11.5)	1		1	
EPTB	652	36 (5.5)	0.96 (0.65 to 1.41)		0.92 (0.36 to 2.32)		87 (13.3)	1.19 (0.90 to 1.56		1.03 (0.84 to 1.27)	
EPTB only^1^	546	20 (3.7)	0.60 (0.37 to 0.99)	0.044	0.58 (0.30 to 1.13)	0.12	74 (13.6)	1.21 (0.91 to 1.61)	0.19	1.04 (0.87 to 1.71)	0.69
Mixed PTB/EPTB^a^	106	16 (15.9)	3.03 (1.74 to 5.29)	<0.001	2.59 (1.06 to 6.32)	0.037	13 (12.3)	1.07 (0.59 to 1.96)	0.81	1.03 (0.62 to 1.71)	0.92
Organs involved^b^				0.72		0.67			0.14		<0.001
Lungs	1772	116 (6.5)	1		1		207 (11.7)	1		1	
Meninges	43	1 (2.3)	1.71 (0.60 to 4.89)		1.76 (0.46 to 6.67)		10 (23.3)	2.28 (1.10 to 4.69)		1.85 (1.00 to 3.10)	
Miliary	26	2 (7.7)	1.39 (0.32 to 5.98)		1.52 (0.38 to 6.02		2 (7.7)	0.63 (0.15 to 2.67)		0.74 (0.31 to 1.74)	
Other	449	18 (4.0)	1.12 (0.73 to 1.68)		1.10 (0.36 to 3.41)		62 (13.8)	1.05 (0.77 to 1.42)		0.87 (0.64 to 1.17)	

ART, antiretroviral therapy; BMI, body mass index; 95% Cl, 95% confidence interval; ART, antiretroviral therapy; EPTB, extrapulmonary tuberculosis; LTFU, lost to follow‐up; OR, odds ratio; PTB, pulmonary tuberculosis; TB, tuberculosis.

The main logistic regression model was adjusted for age, sex, BMI at start of TB treatment, previous history of TB, CD4 cell count at TB treatment start, ART status at TB diagnosis, and site of disease, taking into account heterogeneity across regions (clustering by treatment programmes). The model was based on 2340 patients since patients with TB treatment outcome defined as “not evaluated” (n = 355) were excluded from the analysis. The reference category is indicated with 1.

^a^These estimates were obtained from a separate model (n = 2340) comparing PTB versus EPTB only and mixed PTB/EPTB and was adjusted for age, sex, BMI at start of TB treatment, previous history of TB, CD4 cell count at TB treatment start, ART status at TB diagnosis, and taking into account heterogeneity across regions (clustering by treatment programmes). The reference category is indicated with 1; ^b^these estimates were obtained from a separate model (n = 2340) comparing involved organs lungs versus meninges, miliary, and other organs and was adjusted for age, sex, BMI at start of TB treatment, previous history of TB, CD4 cell count at TB treatment start, ART status at TB diagnosis, and taking into account heterogeneity across regions (clustering by treatment programmes). The reference category is indicated with 1.

The lymph nodes, pleura, abdomen, and meninges were the most frequently involved organs in this large, multicohort study of HIV‐positive EPTB patients treated in ART programmes in sub‐Saharan Africa, Asia‐Pacific, and Caribbean, Central and South America. Diagnosis were mainly based on clinical criteria, and bacteriological confirmation was seen less frequently in EPTB than PTB patients. Mortality was reduced among EPTB patients and PTB patients with a positive diagnostic test result compared to all other TB patients with a negative result. The observation that CD4 cell count in patients with EPTB was frequently lower than that of PTB patients was similar to the report of a South African study, that found that EPTB was generally more common in HIV‐positive patients with lower CD4 cell counts, and three times more frequent among those with HIV and a CD4 count <50 cells/μL than among HIV‐negative individuals 4. Similarly, the predominating involvement of the lymph nodes that we observed is consistent with previous publications on the presentation of EPTB in HIV‐positive adults 19, 20, 21, 22, 23, 24.

The diagnosis of TB is more challenging in HIV‐positive than in HIV‐negative patients. In PTB, this is due to reduced lung cavitation and lower bacterial load in sputum 25, 26, 27, 28. In line with previously published results, we observed that smear microscopy was the most commonly used diagnostic tool, even when other diagnostic modalities were available 16. We further observed that bacteriological confirmation (positive smear microscopy, culture or Xpert MTB/RIF result) was associated with reduced mortality in PTB and EPTB patients compared to TB patients with a negative result. A study from Malawi showed similar results, but also found increased mortality among EPTB patients with a smear‐negative result 29. A systematic review explained the reduced mortality in bacteriologically confirmed PTB cases by showing that smear‐ and culture‐negative disease is typical of advance HIV immunosuppression compared to smear‐positive TB patients with a less compromised immune system 30.

Among the EPTB patients for whom a bacteriological confirmation test was performed, only 42% were confirmed positive. Bacteriological confirmation is challenging due to EPTB's paucibacillary nature, in tissue, body fluid, or cerebrospinal fluid and the need for invasive specimen collection for microbiological diagnosis by biopsy or fine needle aspiration 7, 8, 9. Mycobacterial culture and Xpert MTB/RIF have been shown to reliably diagnose EPTB, but are still rarely used in resource‐limited settings, even when available 13, 16. From a programmatic perspective, the introduction of new diagnostics can indeed increase the proportion of bacteriological confirmed TB patients, as shown by a study from Cape Town, South Africa 31, but this may depend on the clinical setting 32. The newly developed, next generation Xpert MTB/RIF Ultra assay has a higher sensitivity and similar specificity than the first generation Xpert MTB/RIF assay 33, and seems to be particularly useful in EPTB and paediatric TB 34.

While overall mortality was similar in PTB and EPTB patients, the mortality of HIV‐positive patients with TB meningitis was greater than that of HIV‐positive patients with PTB. This is in accord with a review that reported mortality up to 69% for TB meningitis in low‐income countries 6. We observed no difference in the odds of being LTFU during treatment among PTB compared to EPTB patients, which is in line with a Nigerian study 35. However, we found that patients with only EPTB showed lower and mixed PTB/EPTB cases slightly increased risk of LTFU compared to PTB. This could be explained by the fact that the patients with mixed PTB/EPTB diagnosis are too sick to return to the clinic, or might even have died at home. A recent study from Botswana also reported an increased risk for LTFU during treatment in EPTB patients (including mixed PTB/EPTB cases) compared to PTB patients 36.

The main limitation of our study was its potential for misclassification bias. EPTB might have been underestimated due to the limited availability of diagnostic capacities 16 and the clinical practice of not pursuing further diagnoses once a PTB diagnosis has been established. Another limitation of the study was the heterogeneity of the participating ART programmes in terms of TB and HIV treatment, availability of supportive care 16 as well as lack of data on opportunistic infections other than TB. Further analysis of treatment outcomes by diagnostic method was not possible due to the small numbers. Although we could not assess the vital status of those LTFU, the sensitivity analysis that we conducted to assess the potential impact of misclassification of death as lost to follow‐up did not show any differences in the main outcomes. In spite of these potential limitations, our study provides important evidence on the limited diagnostic capacities available for EPTB at ART programmes in LMIC, and is one of few studies investigating EPTB in this context.

## 4 Conclusions

Diagnosis of EPTB in ART programmes in LMIC is based mainly on clinical symptoms, and the introduction of molecular assays is still challenging despite major efforts. We conclude that greater access to diagnostic services could improve diagnosis, increase the number of diagnosed EPTB and improve clinical management of EPTB as well as treatment outcomes.

## Competing interests

All authors have no competing interests.

## Authors’ contributions

KZ, MB, ME and LF involved in conception and design. KZ and MB analysed the data. KZ and LF completed the final draft of the manuscript. MB, SK, HC, MY, BG, DM, TRS, KMN, AMM, ACP and ME provided input into the study design, analyses and drafting of the paper. All authors reviewed and approved the final version of the manuscript.
